# B. M. A. v. L. C. C.

**Published:** 1911-07-08

**Authors:** 


### B. M. A. v. L. C. C.

Whether the party system and the party game as at present flourishing in this kingdom make for good or evil is a question about which opinions may legitimately differ. But it is fairly common ground that the medical profession, as a profession, should hold aloof from politics, although its members as individuals rightly please themselves as to their views and votes on matters of public interest. It is therefore with regret and misgiving that we have read the newspaper summary of the interview between the President of the Board of Education and the deputation from the British Medical Association which waited on him with respect to the inspection of school children in London. The members of the deputation included several of the most extreme Radical politicians in London, and their two spokesmen were both candidates for Parliament in the interest of that party at the last election. The tone they adopted was one of undisguised hostility to the municipal reform majority of the London County Council, and their speeches read more like an attempt to play the party game than to benefit the school children. If these extremists have managed to capture the machinery of the British Medical Association in London for the advancement of their own political views, the fact redounds to their ingenuity and energy; but it is distinctly unfortunate for the Association.

The most extraordinary feature of the whole interview was that Mr. Runciman was actually forced into the position of defender of the London County Council, so violent was the attack upon that body by the spokesmen of the British Medical Association. The position of the former body is quite clear and defined, though the extremists who composed this deputation do not seem to understand it. Saddled with an expensive task by Act of Parliament, and harassed by a Government which is unsympathetic politically, the County Council protests against being compelled to carry on the medical inspection of school children entirely out of local funds, and maintains that the Imperial Exchequer should at least ease the burden. Entrusted at two successive elections with the task of rehabilitating London finance and checking extravagant expenditure, the Council finds every effort at economy opposed and thwarted by the Ministry. Accordingly it declines to shoulder the expensive experiment of starting on an heroic scale free treatment for the children of London, which is quite optional under the Act. The Board of Education has already come into serious collision with the Council, but it naturally shirks the attempt to force the latter to start a hundred clinics which are not compulsory. The President of the Board was obliged to point out to the hotheads that the London County Council employs on the administrative side of the work four times as many doctors as the whole of the rest of England and Wales, and that a hundred medical men are now working as inspectors. It is with reluctance that we feel constrained to pass these criticisms on the deputation and on the action of the Association in sending it. But the best of cases may be spoilt by exaggeration, and the most important schemes of national hygiene must eventually be based upon sound finance. By ignoring these two facts the Association has made itself ridiculous, and has increased the suspicion of those who regard it as a narrow trade-union run in the financial interest of the medical profession but professing nobler and less selfish objectives.

